# Beyond the Conventional: PsyQ – a Space for Curiosity, Collaboration, and Cultural Sensitivity in Psychiatry

**DOI:** 10.1192/bjo.2025.10292

**Published:** 2025-06-20

**Authors:** Sanaa Moledina, Seema Uchil, Harshani Yapa Bandara, Faquiha Muhammad

**Affiliations:** Northamptonshire Healthcare NHS Foundation Trust (NHFT), Northampton, United Kingdom

## Abstract

**Aims:** Traditional psychiatry training often prioritizes service delivery, limiting reflection and exploration. Few opportunities for intellectual discussions outside academic meetings lead to monotony and rigidity in practice. Poor networking opportunities can be isolating, especially for those unfamiliar with the UK’s multicultural landscape.

PsyQ was created by doctors at Northamptonshire Healthcare NHS Foundation Trust (NHFT) to overcome these gaps. The platform aims to nurture curiosity, cultural sensitivity, and receptiveness among doctors by offering a relaxed, judgment-free space to engage in meaningful discussions. The topics covered are wide-ranging, spanning psychiatry’s intersections with disciplines like philosophy, religion and spirituality, law, and social sciences, as well as issues in the contemporary social milieu.

**Methods:** Launched in March 2024, PsyQ has hosted discussions on a wide array of topics, including evolutionary psychiatry, the assisted dying bill, sexual orientation and the nature versus nurture debate, patient confidentiality, the experience of immigrant doctors in the UK, free will, the role of psychiatrists in preventing death, and the psychological concept of safety. To assess the platform’s impact, an electronic feedback form was distributed. Fifteen participants completed the form. The survey combined Likert-scale with open-ended questions to evaluate overall participants’ experience, inclusivity, topic relevance, session engagement, and suggestions for improvement.

**Results:** The results highlighted PsyQ’s success in creating an inclusive and engaging environment. Eighty per cent of respondents rated their overall experience 4/5 or higher, while 78% found the topics highly relevant to their practice and daily lives. The platform’s relaxed and inclusive atmosphere stood out, with 85% of attendees commending its openness. Additionally, 75% described the format as highly effective, citing the balance between structured discussions and open-ended dialogue as conducive to exploring diverse viewpoints. PsyQ significantly influenced attendees, with 90% reporting that sessions challenged their thinking, 85% feeling encouraged to tolerate differing opinions, and 70% gaining practical insights applicable to their work. Participants valued the diversity of topics, the safe space for discussing sensitive issues, and networking opportunities. Suggestions for improvement included adjusting session timings, extending session lengths, and securing funding to enhance the overall experience.

**Conclusion:** PsyQ is a platform that allows doctors to network, think creatively, express opinions, and gain new insights. It is crucial for doctors to be flexible, culture-aware, and well-informed of broader societal perspectives. Moving forward, the programme plans to incorporate participant input, expand its range of topics and guest speakers, and develop strategies to measure its long-term impact, ensuring continued adaptability to participant’s needs.

